# Urgent need to address increasing caesarean section rates in lower-middle-income countries like Bangladesh

**DOI:** 10.3389/fgwh.2024.1365504

**Published:** 2024-07-12

**Authors:** Monaemul Islam Sizear, Mamunur Rashid

**Affiliations:** ^1^ Technical Advisor, Health Systems for TB, Open Development and Organizing Secretary, Public Health Foundation, Dhaka, Bangladesh; ^2^ Unit of Public Health Sciences, Faculty of Health and Occupational Studies, University of Gävle, Gävle, Sweden

**Keywords:** cesarean section, pregnancy, maternal health, lower-middle-income countries, Bangladesh

## Introduction

Cesarean section (CS) delivery has substantially increased globally over the past few decades (1). The prevalence of CS worldwide has increased from about 16 million in 2000 to 29.7 million in 2020 (2). Data from 2010 to 2018 covering 154 countries and a total of 94.5% of world live births show that 21.1% of women have given birth by CS (3). This escalating trend has been observed in all regions, particularly low- and middle-income countries from Eastern and Southern Asia and Northern Africa, indicating a sharp increase of 44.9% and 31.5%, respectively, and the trend has emerged as a critical global health concern (3). The prevalence of CS increased by 27% in Bangladesh during the past decade (i.e., 18% in 2011 to 45% in 2022) (4), which is a much higher figure compared to neighboring countries such as India (14%), Pakistan (14%), and Nepal (4%) (5). According to the World Health Organization (WHO) (6), CS rates between 10% and 15% are considered an acceptable standard. This means that CS rates in Bangladesh are threefold higher than WHO’s acceptable standard, which is alarming for maternal health, newborns, families, and the healthcare system. More specifically, the CS rate in Bangladesh is higher among mothers who live in urban areas (36.9%) than among mothers who live in rural areas (17.9%) (7). Regarding divisional levels, the rate of CS delivery is the highest in Khulna (35.2%), followed by Dhaka (32.9%), Rajshahi (28%), Chattogram (20.1%), Barishal (19.90%), Rangpur (19.50%), and Sylhet (13.30%) (7).

The present opinion article aims to explore the primary reasons for the dramatic increase in CS delivery in Bangladesh and proposes evidence-based recommendations for reducing the rate of unnecessary CS.

## Discussion

### Factors affecting increasing CS rates in Bangladesh

Despite enormous improvements in maternal and child health in Bangladesh over the years, CS rates have risen to unprecedented levels in both rural and urban areas (2, 8). There are two main reasons for CS: (i) medical necessity, meaning CS is required to save a life, and (ii) elective surgery, meaning an unnecessary CS that is usually performed to control delivery. One of the potential reasons for the high rate of CS deliveries in Bangladesh may be elective CS, which is associated with a tendency toward profit-making. One previous study reported that physicians and the breadwinner of the pregnant woman’s family are the primary decision-makers behind CS delivery and that pregnant women are often excluded from that decision-making process (9). Thus, women are probably not aware that an unnecessary CS can result in increased risk of infection, potential complications in subsequent pregnancies, and prolonged recovery times (10). Previous evidence has shown that a combination of sociocultural factors, such as education, social class, and religion, and medical factors, such as pregnant women’s high blood pressure and/or diabetes, contributes to this increasing CS rate (2). Research has also indicated that, although protocols and guidelines for CS are available in healthcare facilities, they are not being followed due to pressures from external agents who are present in public hospitals and who want to benefit financially by facilitating the transfer of patients to private hospitals (11).

Given the different reasons for CS, it is also important to mention that private healthcare facilities contribute to the greater part of CS in Bangladesh (12). Approximately 3.6 million babies are born each year in Bangladesh, and 50% of deliveries are institutional. Among these deliveries, two-thirds occur in private facilities (83% private facilities vs. 35% government facilities) (13). The financial benefits gained by private hospitals constitute the main reason for encouraging pregnant women and their families to undergo CS delivery rather than examinations of pregnant women’s physical condition (7, 10). One study claimed that highly educated women and their husbands tend to prefer CS without hesitation compared to less educated couples, especially in urban areas (5). One reason could be that educated couples have no economic barriers, meaning they can avoid vaginal delivery complications without being informed about the consequences of an unnecessary CS.

### Evidence-based policy formulation and imperative action

Considering the physical and financial burdens of unnecessary CS, evidence-based recommendations could help change the scenario in Bangladesh. One study recommended a health reform to reassess the role of private healthcare facilities and redefine the responsibilities of private healthcare providers (14). Another study from Iran stressed that, while reducing CS rates is complex and time-consuming, key factors include informing mothers and families about the benefits of normal delivery, strong government regulations, and strict supervision of healthcare workers (15). A study from Brazil suggested that improving healthcare facilities, including hospital electronic medical records, data management systems, and standardized prescription systems, aids in making informed decisions, thereby increasing the rate of vaginal births in private healthcare facilities from 2010 to 2015 (16). A global systematic review found that, in some countries, financial incentives for service providers and legislatively imposed practice guidelines for physicians could control CS rates to some degree (17). These studies mentioned that reducing CS rates cannot be achieved through a single, quick intervention. Instead, achieving effective outcomes requires a comprehensive, context-specific approach and considerable time. Additionally, a systematic review strongly suggested the need for regulatory strategies aimed at reducing unnecessary CS procedures that could be effective across both public and private institutions (17). There is also evidence indicating that responsible medical leadership is crucial in reducing unnecessary CS procedures, alongside the introduction and implementation of strong regulations (3).

To reduce unnecessary CS rates, the government could track and report the annual rate of unnecessary CS and its costs. Including this information in the policy-making process could play a pivotal role in building awareness about unnecessary CS delivery. Increasing the number of trained midwives serving across the country is important to ensuring that unnecessary CS can be avoided, unless of course pregnant women experience physical complications (2). It is also necessary to involve pregnant women in the decision-making process and inform them of the benefits and risks of a CS (10). Moreover, community-level awareness programs and proper sensitization of mothers and families should continue to enhance their knowledge of the implications of unnecessary CS deliveries (5). Additionally, adopting a global standard like Robson’s classification system, which categorizes CS cases based on obstetric parameters such as pregnancy history and gestational age, could be incorporated into policies and regulations to help midwives and physicians make the right decisions (6, 18). The government could incorporate appropriate guidelines for CS into policy and regulations to ensure that no hospital, whether public or private, performs unnecessary CS procedures (5, 11). Healthcare regulatory bodies could develop a national guideline for use of CS that healthcare providers are obliged to follow.

WHO recommends CS delivery based on the mother’s physical condition and the position of the fetus, but these recommendations are not always followed by healthcare providers in Bangladesh. In this regard, the government could expand social and community movements to create awareness, particularly among pregnant women, of the negative immediate and long-term consequences of unnecessary CS delivery. Awareness and education efforts could also be focused on the benefits of vaginal delivery, such as faster recovery, low cost, and benefits to the baby (17, 19). However, CS can be used to save lives in certain cases, for instance, to deal with adverse situations like uterine rupture, pelvic pain, abnormal placentation, ectopic pregnancy, and many more (5, 8, 20). Based on the above opinion and evidence-based statement concerning why the rate of CS is increasing rapidly, particularly in Bangladesh, a tailored strategy that considers the different perspectives of healthcare providers and pregnant women and that utilizes the Robson Ten Group Classifications could be applied to reduce unnecessary CS.

## Conclusions

Although CS has emerged as a life-saving intervention, the rate of unnecessary CS has increased over the years, particularly in low-middle-income countries, including Bangladesh. The Bangladeshi Government is committed to reducing the maternal mortality rate and ensuring universal access to reproductive healthcare to attain the Sustainable Development Goals in this regard, but the rising CS rates negatively affect maternal and reproductive health and could hinder achieving these SDGs by 2030. Moreover, the total out-of-pocket cost due to CS is around ten times higher at the facility level compared to home delivery, and this additional cost has a compound effect on the country’s healthcare system and access to quality maternal care (18). Therefore, it is pivotal to understand the facts related to medical necessity, patient autonomy, and respectful maternity care for all women, the goal being to avoid unnecessary CS before the practice becomes even more widespread. Because the factors influencing CS constitute a complex phenomenon, further studies are needed to understand the issue from different perspectives – such as those of healthcare providers, pregnant women, family members – and in relation to community norms and practices. Qualitative studies are of particular importance in this context.
